# A Resistive Soft Robotic Exosuit for Dynamic Body Loading in Hypogravity

**DOI:** 10.1002/advs.202506057

**Published:** 2025-09-30

**Authors:** Emanuele Pulvirenti, Richard Suphapol Diteesawat, Gaspare Pavei, Valentina Natalucci, Helmut Hauser, Alberto Minetti, Jonathan Rossiter

**Affiliations:** ^1^ School of Engineering Mathematics and Technology University of Bristol Bristol BS8 1TW UK; ^2^ Bristol Robotics Laboratory Bristol BS16 1QY UK; ^3^ Department of Pathophysiology and Transplantation University of Milan Milan 20133 Italy

**Keywords:** Soft Exosuits, Health in Space, Hypogravity, Soft Robotics, Human spaceflight, Muscle activation

## Abstract

Prolonged exposure to reduced gravity in space leads to bone demineralization and muscle atrophy, which current countermeasures of simple body loading can partially address. To address this aetiology, a resistive hypogravity exosuit (R‐HEXsuit) is proposed for dynamic body loading in low gravity. R‐HEXsuit is a lightweight (1.4 kg) soft wearable exosuit that uses pneumatic artificial muscles to provide programmed bilateral resistance during walking, stimulating primary leg muscles. Tests on healthy subjects in Earth gravity and simulated Moon gravity reveal that the suit increases metabolic cost by 29.3% in Moon gravity, aligning it with Earth‐like metabolic cost. Muscle activation in key knee joint muscles also increases, matching or exceeding Earth levels, without altering natural gait patterns. These results highlight the R‐HEXsuit as a promising tool for replicating Earth‐like physical demands during low‐gravity missions, offering a potential solution for mitigating musculoskeletal degradation in space.

## Graphical abstract

The resistive hypogravity exosuit is a lightweight wearable robotic exosuit that uses pneumatic artificial muscles to restore Earth‐like metabolic and muscular demands during locomotion in low gravity. Tested on healthy subjects in Earth and simulated Moon gravity, the suit restores energy expenditure and muscle activation without altering gait, offering a potential additional countermeasure against musculoskeletal decline in space.

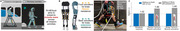

## 1 Introduction

As space becomes more accessible, with long missions planned to the Moon and Mars, maintaining the health of humans remains a major challenge. In particular, prolonged exposure to hypogravity (gravity lower than Earth's) has a detrimental effect on the musculoskeletal health of astronauts.^[^ ^1^ ^]^ It has been shown that crewmembers returning from the International Space Station (ISS) exhibit significant performance decline in tests requiring dynamic postural control.^[^ ^2^ ^]^ The reduced health has been attributed to sarcopenia (loss of muscle mass) and bone demineralization, both associated with the reduced mechanical loads of lower‐than‐Earth gravity. Bone demineralization occurs at a rate of 1%–2% of bone mass per month of space flight, especially in the weight‐bearing regions (lower limbs) of the body.^[^ ^3^ ^]^ This greatly increases risk of fracture and premature osteoporosis.^[^ ^4^ ^]^ Additionally, losses of up to 20% of muscle mass and up to 30% of muscle strength per month have been noted in astronauts exposed to microgravity.^[^ ^4^, ^5^ ^]^ Decreases in muscle volume, peak force, and contraction velocity have also been observed in space flight returnees.^[^ ^6^ ^]^ As a result, postflight rehabilitation can take from several weeks to several months, with a large intersubject variability.^[^ ^7^ ^]^

**Figure 1:**
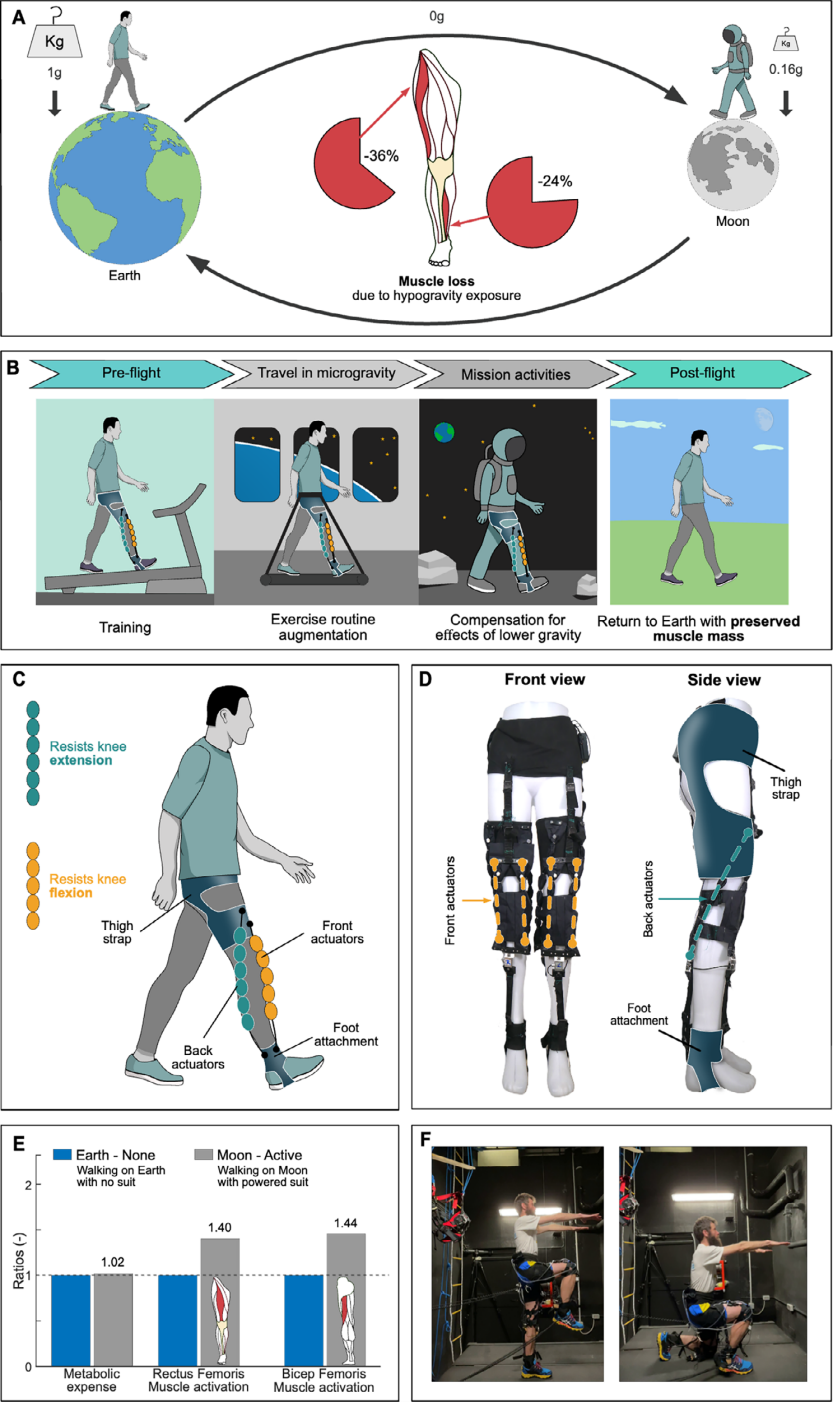
Soft Resistive Hypogravity Exosuit (R‐HEXsuit) for dynamic body loading in hypogravity. A) Effects of long‐term exposure to hypogravity, include muscle atrophy (sarcopenia), mostly on the lower limb muscles, with vastus lateralis (quad muscle) and soleus (calf muscle) losing 36% and 24% of their cross‐sectional area after 11–17 days in hypogravity.^[^ ^3^, ^4^, ^5^, ^12^, ^21^ ^]^ B) The R‐HEXsuit and envisioned uses. The R‐HEXsuit can simulate Earth‐like loading on astronauts’ lower limbs, by providing resistance to their movement. The R‐HEXsuit has potential to enhance the preflight training, augment the exercise regime during microgravity transfer, simulate Earth‐like loading during daily tasks in the Moon habitat, and during extravehicular activities. C) Diagram of the R‐HEXsuit and main components. D) Front and side views of the R‐HEXsuit prototype used during simulated low gravity testing. E) Impact of suit resistance in simulated Moon gravity (Moon‐Active), causing increase in metabolic cost and muscle activation of the rectus femoris (RF) and bicep femoris (BF). The results are presented as ratios relative to the Earth–Nonecondition (not wearing the suit). Earth values are depicted with blue bars, while Moon values are shown with gray bars. F) Demonstration of unaffected range of motion while wearing the R‐HEXsuit.

As major international space agencies plan for human settlements on the Moon, addressing the adverse effects of hypogravity becomes critical (**Figure** **1**A). Unlike short‐term ISS missions, future lunar astronauts will face extended stays, potentially lasting a year or longer,^[^ ^8^ ^]^ which require adaptation to prolonged low‐gravity (0.16 g) exposure. This extended time in hypogravity will necessitate more rigorous countermeasures to prevent musculoskeletal injuries and support postmission rehabilitation. Additionally, the Lunar Gateway, serving as a staging point for lunar surface missions, will expose astronauts to microgravity during transit, further complicating the physiological adaptations as they move between Earth's gravity, the Gateway's microgravity, and the Moon's reduced gravity. While lunar missions are the immediate priority, plans for human missions to Mars introduce even greater challenges. Mars has a higher gravity (0.38 g) than the Moon but will involve longer transit times, with astronauts potentially spending up to 540 days in microgravity flight and several months on Mars.^[^ ^1^ ^]^ This constant shift between gravitational environments, from Earth to space to planetary surfaces, heightens the risk of injury, and emphasizes the need for advanced countermeasures. In this work, we will focus on the effects of Lunar gravity.

Existing measures to counter the deleterious physiological effects of low gravity depend on pharmaceutical interventions^[^ ^9^ ^]^ and a rigorous daily exercise regimen.^[^ ^10^ ^]^ Despite showing some level of mitigation, positive effects have been limited and variable across subjects.^[^ ^3^, ^11^, ^12^, ^13^ ^]^ For this reason, alternative and complementary approaches are being explored to maintain astronaut health in hypogravity, particularly through powered exoskeletons, which have been extensively used in rehabilitation on Earth.^[^ ^14^ ^]^ NASA considered the employment of the X1 exoskeleton,^[^ ^15^ ^]^ developed in 2013, in conjunction with the exercise apparatus present on the ISS. The Human Universal Load Carrier (HULC), another exoskeleton developed in 2020, was also considered for use in long‐term missions to the Moon.^[^ ^16^ ^]^ However, both are heavy and bulky (with reported mass of 27.2 kg for the X1^[^ ^15^ ^]^ and 24 kg for the HULC^[^ ^16^ ^]^). Generally, rigid exoskeletons are expected to be difficult to integrate into pressurized spacesuits, where limited internal volume and joint misalignment have been linked to shoulder injuries and mobility issues during EVA operations.^[^ ^17^, ^18^ ^]^ Rigid exoskeletons have also been shown to be uncomfortable to wear for long periods^[^ ^19^, ^20^ ^]^ and have not been employed in space applications.

Recent advances in soft robotics, especially in high power density actuators, have enabled soft wearable robots and soft powered exosuits to become viable alternatives to traditional, rigid, electrically‐driven exoskeletons.^[^ ^22^ ^]^ Exosuits are naturally compliant and lightweight, providing a safer physical interface with the user and enabling a more natural range of motion.^[^ ^20^ ^]^ They can deliver power to the wearer while offering a comfortable fit similar to clothing. While some exosuits require additional components like air pumps, valves, and pressurized air supplies, overall they remain significantly lighter and less bulky than their rigid counterparts. They are also typically easily manufacturable using low cost, rapid, and proven textile processing techniques including laminating, embroidery, screen printing, heat sealing, and sewing.^[^ ^23^ ^]^ More broadly, recent advances in fabric‐based pneumatic actuators have further strengthened the case for textile‐integrated wearables, highlighting their adaptability, manufacturability, and integration with sensing textiles.^[^ ^24^ ^]^ Additionally, it has been demonstrated that actuator enclosure and anchoring strategies significantly affect performance in exosuits.^[^ ^25^ ^]^ These developments show how textile integration can be optimized for reliability and comfort. In parallel, recent developments in inflatable and textile‐driven wearable robots^[^ ^26^, ^27^ ^]^ highlight how pneumatic and sensor‐integrated exosuits are reaching practical maturity, demonstrating compact, functional designs suitable for real‐world applications. Collectively, these advancements demonstrate that soft wearable systems are progressing from assistive prototypes to robust textile platforms.

Soft exosuits have been successful in delivering body assistance and reducing the metabolic cost of walking,^[^ ^28^, ^29^, ^30^ ^]^ for neurological and poststroke rehabilitation,^[^ ^31^, ^32^ ^]^ and in industrial applications.^[^ ^33^ ^]^ In the last decade, there has also been a shift toward the development of less bulky and more compliant spacesuits, exemplified by the BioSuit.^[^ ^34^ ^]^ This trend has led to the emergence of soft wearable devices that can be easily integrated with new types of spacesuits to mitigate the harmful effects of long‐term exposure to microgravity.^[^ ^35^, ^36^, ^37^ ^]^

Previous soft exosuits were conceived primarily to assist the motion of the user against the loading of Earth's gravity, with the intention to decrease the metabolic cost of certain activities (e.g., a soldier carrying a heavy pack) or to increase the wearer's strength or mobility (e.g., to support people with disabilities). In contrast, one of the core challenges to extraterrestrial living is the lack of body loading arising from low gravity.^[^ ^11^ ^]^ If Earth‐like loading could be imposed on astronauts in space, for example through a controllable and wearable resistive device, muscle strength would be maintained and tissue degeneration would be reduced.^[^ ^38^ ^]^ This has been the main requirement for the development of exercise hardware on board the ISS.^[^ ^39^, ^40^ ^]^

Here, we present a first‐generation pneumatic Resistive Hypogravity Exosuit (R‐HEXsuit), fabricated from textile materials. The R‐HEXsuit is envisaged as a daily garment across all stages of a long‐term space mission (Figure 1B). Such a device could serve as a complementary countermeasure to resistive exercise, addressing limitations of current equipment like the aRED (advanced Resistive Exercise Device), which has shown limited effectiveness in preventing bone and muscle loss during short‐ and medium‐term spaceflight.^[^ ^12^, ^13^ ^]^ Studies involving 37 ISS crew members showed that the aRED and its predecessor, the iRED, did not fully replicate Earth‐like loading. Even with aRED use, statistically significant losses in isokinetic lower body strength (−4.3% to −14.5%) were observed after missions averaging 163 days. While aRED users tended to experience less strength decline than iRED users, the difference was not statistically significant, underscoring the need for more effective or complementary countermeasures.^[^ ^12^ ^]^ Furthermore, the aRED is bulky, fixed in place, and used only during scheduled exercise sessions. Other studies have highlighted the value of exercise equipment that is lightweight, self‐powered, capable of providing Earth‐like loading, effective with relatively short exercise durations, and minimally disruptive to the surrounding environment in terms of noise or structural vibrations.^[^ ^41^ ^]^ Hence, the R‐HEXsuit was developed to be lightweight, wearable, and capable of providing Earth‐like resistance throughout routine intravehicular activity. As such, we hypothesize that it could offer a continuous, low‐disruption stimulus that complements structured exercise sessions by extending loading throughout the day.

The current prototype exosuit (Figure 1C,D) is flexible and conforms to the user's body, enabling a wide range of movements (Figure 1F). The R‐HEXsuit offers active resistance that stimulates the body's muscles to activate to Earth‐like levels, tailored to the user's walking gait. It employs lightweight textile‐based pneumatic Bubble Artificial Muscles (BAMs) that have a high strength‐to‐weight ratio and are capable of dynamically delivering suitable force and stroke for wearable applications at low operating pressure.^[^ ^42^, ^43^ ^]^

The functionality and effects of the R‐HEXsuit were evaluated through human trials in a hypogravity analog environment at the L.O.O.P. (Locomotion On Other Planets) ESA (European Space Agency) Ground Based facility of the University of Milan. This facility was chosen as one of the standard methods of investigating the effects of simulated low gravity on human physiology through weight offloading.^[^ ^44^, ^45^, ^46^, ^47^ ^]^ Other methods for simulating reduced gravity on Earth, such as parabolic flights and vertical or tilted treadmills,^[^ ^48^, ^49^ ^]^ were considered but deemed unsuitable for this study. Parabolic flights, while effective for short (less than a minute) simulations, offer limited duration in low gravity, which prevents performing steady‐state metabolic investigations. Vertical treadmills, on the other hand, rely on bulky support exoskeletons, which restrict space and make the implementation and testing of the exosuit impractical. More details on the L.O.O.P. facility can be found in the Experimental Section. It is important to note that, although this approach mimics low gravity by reducing the body's net weight through the application of constant upward force, all parts of the body are still exposed to gravity. For example, the dynamics of leg swing remain influenced by Earth's gravity.^[^ ^48^, ^50^, ^51^ ^]^

### 1.1 Working Principle of the R‐HEXsuit

**Figure 2:**
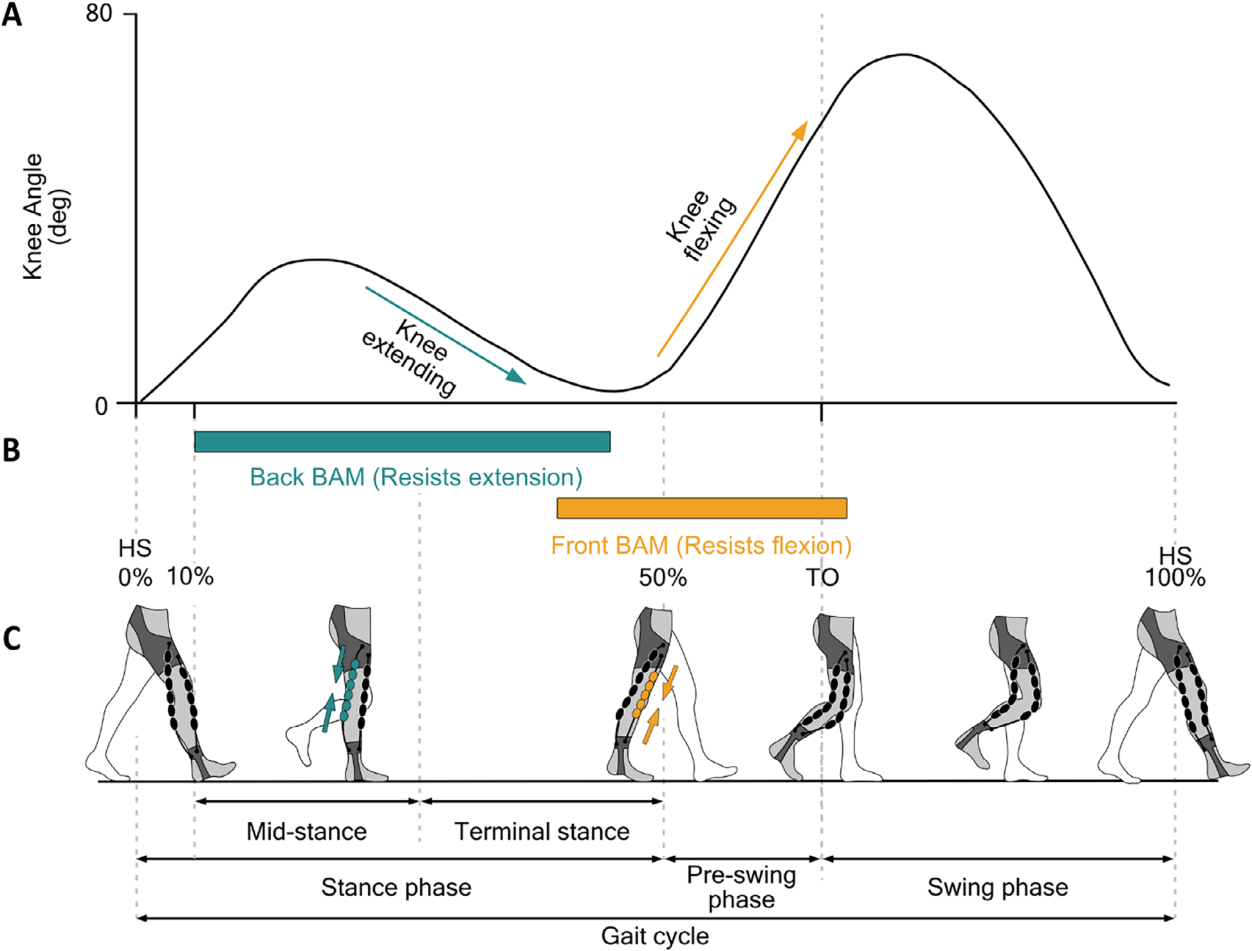
The application of the resistance delivered by the R‐HEXsuit during one gait cycle. A) Typical knee angle trajectory. Reproduced with permission.^[^ ^52^ ^]^ Copyright David Delp, 2020.; arrows indicate knee extending and flexing. B) Timing of applied resistance on the knee extension and flexion, applied through the actuation of the back BAMs and the front BAMs, shown as green and orange bars, respectively. C) Diagram illustrating different phases of the right leg (wearing the suit) during a single gait cycle, where HS indicates heel strikes, and TO indicates toe‐off. The cycle starts at the first HS and ends at the second HS as 0% and 100% of the gait cycle, respectively.

A typical walking gait can be divided into three phases: stance, preswing, and swing (**Figure** **2**C). During the stance phase (from the first heel strike (HS) at 0% to ≈50% of gait cycle), knee muscle flexors (initially) and extensors (subsequently) contribute to body weight loading, single‐leg support, and body propulsion.^[^ ^52^ ^]^ At ≈50%, the gait enters the preswing phase, with the initiation of knee flexion, employing knee muscle flexors and the muscles around the ankle (calf muscles and tibialis), preparing for the swing phase. The swing phase initiates with toe off (TO) at ≈60% of the gait cycle and continues to the end of the cycle at 100% and the next HS of the new cycle (0%). The actuation of the R‐HEXsuit was tailored to this Earth‐like gait cycle. While skipping and hopping have been shown to be more economical in terms of metabolic cost on the Moon at intermediate and high speed, studies suggest that walking remains more economical at speeds below 1.4 m s^−1^.^[^ ^44^ ^]^ Therefore, for the walking speed selected in this study (1.1 m s^−1^ or 4 km h^−1^), the same gait cycle characteristics can be applied on the Moon.

The R‐HEXsuit applies resistance to knee extension and flexion during the stance and preswing phases respectively, when the leg is in contact with the ground, and relaxes during the swing phase. Two pairs of soft BAM actuators were placed at the front and back of the knee joint, labeled front BAMs and back BAMs. The choice of applying resistance only to the knee joint was based on physiological, biomechanical, and design factors. Previous studies^[^ ^21^, ^53^, ^54^ ^]^ have reported losses of cross‐sectional area of the vastus lateralis (quad muscle) and soleus (calf muscle) of 36% and 24% after 11–17 days in hypogravity. These muscle groups intersect at the knee joint and are responsible for knee extension and knee flexion. These findings highlight the vulnerability of muscle groups spanning the knee joint, as they are heavily involved in load‐bearing and locomotor functions under Earth gravity and are disproportionately affected by unloading in space. Biomechanically, the knee joint undergoes the greatest range of motion during walking, exhibiting large flexion‐extension angles compared to the hip and ankle joints. This makes it an ideal site for introducing dynamic resistive loading, especially in early‐stage prototype development where observable changes in gait and muscle activity can be more readily detected and quantified.

Previous work on assisting knee swing in simulated low gravity^[^ ^55^ ^]^ has shown that delivering assistance at the knee joint yields positive results in terms of improved flexion angle and speeds. From a design perspective, beginning with a single‐joint implementation allowed us to simplify fabrication and control, while still yielding meaningful physiological insights. Targeting the knee thus served as a proof‐of‐concept for demonstrating the feasibility of the resistive actuation approach and its potential to restore muscle engagement in hypogravity conditions.

The placement of the BAMs and the exosuit components are shown in Figure 1C,D. The suit applies resistance to the knee extension through the actuation of the back BAMs from mid to terminal stance (10%–45% of gait cycle; green bar in Figure 2B) and to knee flexion through the actuation of the front BAMs between terminal stance and preswing phase (40%–60% of gait cycle; orange bar in Figure 2B). Resistance was applied in two forms: passively, through the intrinsic elastic properties of the BAMs, while unactuated, and actively, by controlling the inflation of the actuators along the gait cycle as described above. This setup was designed to assess the suitability of, and provide a comparison between, passive and active resistance. The actuation of both BAM actuators and the available range of motion while wearing the R‐HEXsuit are shown in Movies S1 and S2 (Supporting Information), respectively.

The primary objective of this study was to evaluate the capability of the R‐HEXsuit to deliver effective resistance in Moon gravity (0.16 *g*). We hypothesized that applying resistance in Moon gravity can restore metabolic cost and muscle activation to levels observed under Earth gravity, thereby compensating for the expected reduction of these physiological parameters during walking on the Moon.^[^ ^45^ ^]^ We evaluated the effect on metabolic cost and muscle activation patterns caused by the resistance delivered by the suit. To evaluate the resistance performance, three walking conditions were investigated: Walking without the suit (None); Wearing the powered‐off exosuit with passive tension (Passive); Wearing the powered‐on suit with activated resistance (Active). These walking conditions—None, Passive, Active—are applied to the two environments (Earth, Moon) to yield six distinct experimental cases: (Earth‐None, Earth‐Passive, Earth‐Active, Moon‐None, Moon‐Passive, and Moon‐Active).

Through this study, we gained insights into how walking mechanics and activation of the leg muscles are influenced by the R‐HEXsuit in simulated hypogravity, providing important information for future resistive devices and advancing our understanding of mitigating musculoskeletal degeneration.

## 2 Results

We tested the R‐HEXsuit on six healthy participants (*n* = 6) for a 4‐min walking test (requirement for steady‐state metabolic assessments, as shown in refs. [45, 56]) at a standard walking velocity of 4 km h^−1^ (1.11 m s^−1^) for all three walking conditions both in Earth gravity (Earth‐None, Earth‐Passive, Earth‐Active) and in simulated Moon gravity (Moon‐None, Moon‐Passive, Moon‐Active), see Movies S3 and S4 (Supporting Information).

Initially, participants walked without the exosuit in Earth gravity (Earth‐None) while metabolic cost and muscle activation were measured (more details in the Experimental Section). The exosuit was then worn, and the subjects were asked to walk with the exosuit providing passive resistance (Earth‐Passive). The suit was well fitted to the body to avoid slack in the actuators (see section “Fitting procedure of the exosuit” for more details). The passive tension of the actuators was adjusted to reach a maximum of 20 N within the gait cycle. Finally, walking with activation of the exosuit (Earth‐Active) was undertaken (maximum resistive force of 60 N). After that, the participants were asked to walk under the same conditions in simulated Moon gravity (Moon‐None, Moon‐Passive, and Moon‐Active, respectively).

Two primary measurements were taken during the study: 1) metabolic cost, measured by indirect calorimetry, and 2) electromyography activity of seven main muscles involved in walking, principally the extensors and flexors of the knee and ankle joints (see sections “Metabolic rate measurements” and “Electromyography (EMG)” for more details; see Figure S2 (Supporting Information) for a detailed diagram of the investigated muscles). Additionally, we evaluated how wearing the exosuit and its applied resistance affected the participants’ walking patterns by analysing the spatiotemporal characteristics of each participant's gait, i.e., time the user's foot contacts ground, duty factor and stride frequency. Data concerning the analysis of the walking conditions on the Moon are presented in the main text. Metabolic cost and muscle activation data for the Earth case are presented in Figures S3 and S4, Supporting Information (Supporting Information). Metabolic cost was selected as it provides insight into changes in cardiovascular demand, serving as an indicator of exercise intensity. Meanwhile, EMG data were utilized to evaluate the neurological effort required by specific muscles.

### 2.1 Effective Increase in Metabolic Cost

**Figure 3:**
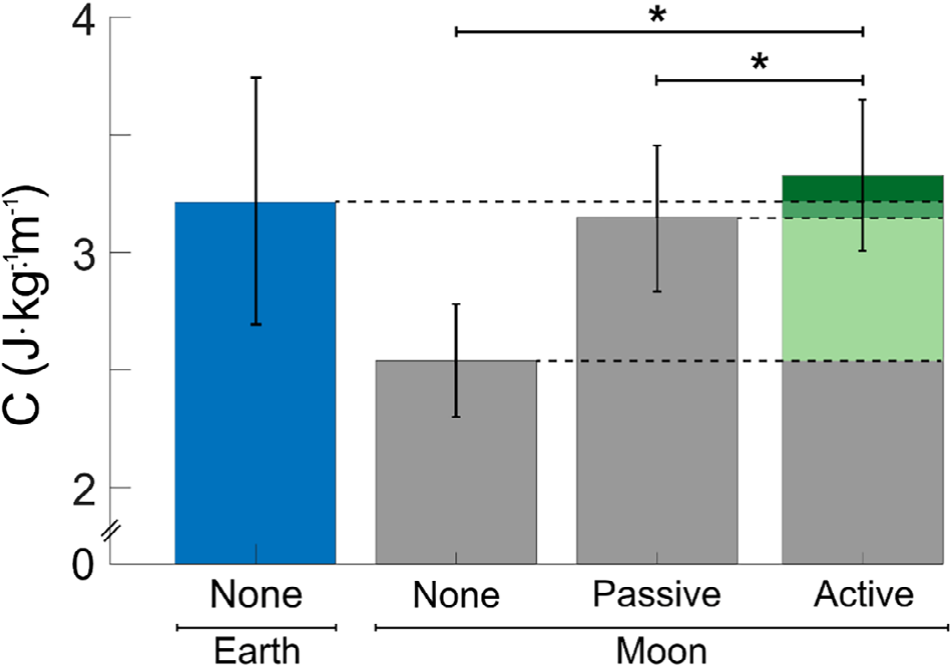
Summary of metabolic cost results. Average metabolic cost measurements for following testing conditions: not wearing the exosuit on Earth (Earth‐None) and on the Moon (Moon‐None), wearing the exosuit without activation on the Moon (Moon‐Passive), and powered exosuit with resistance on the Moon (Moon‐Active). Green shaded areas indicate increments in metabolic cost between conditions, compared to the Moon‐None condition. Asterisks indicate statistically significant results (*n* = 6; *P* = 0.0410, *P* = 0.0165); vertical bars indicate standard deviation.

First, walking conditions on Earth gravity were analyzed. Under Earth gravity (shown in Figure S3, Supporting Information), wearing the exosuit while providing passive resistance (Earth‐Passive) caused a significant increase in average metabolic cost of 18.2% over the Earth‐None condition (*n* = 6, two‐sided paired *t*‐test, Holm–Šidák correction, *P* = 0.0211). When applying active resistance (Earth‐Active), the average metabolic cost rose to 20.1% compared to metabolic cost under the Earth‐None condition (*P* = 0.0111). When comparing walking without the exosuit between Earth and Moon gravity, the Earth‐None condition resulted in 26.9% more metabolic cost than under the Moon‐None condition (*P* = 0.0154) (**Figure** **3**).

When wearing the passive exosuit under Moon gravity (Moon‐Passive), the metabolic cost increased by 20.1% over the Moon‐None condition. When the suit was activated under Moon gravity, i.e., Moon‐Active, the metabolic cost increased notably by 29.3% compared to the Moon‐None condition (*P* = 0.0410) and 7.7% compared to the Moon‐Passive condition (*P* = 0.0165). Notably, the active resistance condition on the Moon (Moon‐Active) resulted in a 1.94% higher metabolic cost than the Earth‐None condition, with no statistically significant difference (*P* = 0.872).

This finding suggests that the resistance provided by the active exosuit (Moon‐Active) can generate sufficient body loading which results in Earth‐like metabolic demands in the simulated lower‐gravity environment of the Moon. This could provide a stimulus which might benefit the astronauts’ musculoskeletal health in the long term. Full metabolic cost comparison between all walking conditions of the Earth and Moon are shown in Table S1 (Supporting Information).

### 2.2 Increase in Muscle Activation of Lower Limb Muscles

**Figure 4:**
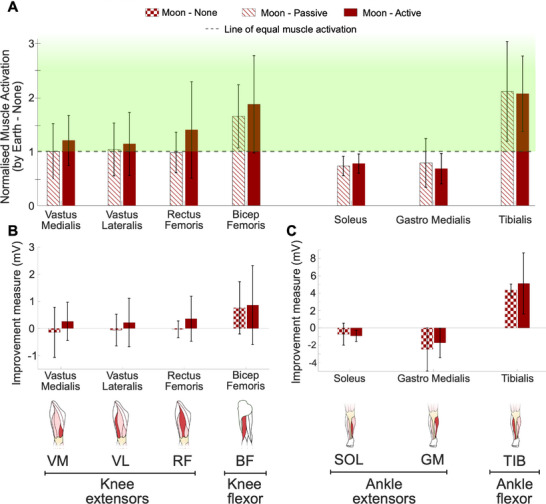
Muscle activation of seven main muscle groups contributing to walking. A) Muscle activation of the Moon‐Passive and Moon‐Active conditions, normalized by the Earth‐None condition. The green region highlights the increase of the muscle activation compared to the Earth‐None condition. (B,C) Differential measurement of the muscle activation of the Moon‐None and Moon‐Active conditions compared to the Earth‐None condition. Vertical error bars indicate one standard deviation. In the Moon‐Passive condition (Figure 4A), the average muscle activation of the knee flexor (BF) and the ankle flexor (TIB) increased considerably, compared to the Earth‐None reference condition (dashed horizontal line). This is partially due to the new simulated gravity environment, which prompts these two muscles to activate more when compared to Earth‐None. The activation of the knee extensors (VL, VM, RF) rose up to the similar muscle activation level of the Earth‐None condition while that of the ankle extensors (SOL, GM) activated less.

In addition to metabolic cost, we investigated the effect of the R‐HEXsuit resistance on the muscle activation of seven key lower limb muscles (**Figure** **4**) known to be contributing to walking: Vastus medialis (VM), vastus lateralis (VL), and rectus femoris (RF) are knee extensors; bicep femoris (BF) is a knee flexor; soleus (SOL) and gastro medialis (GM) are ankle extensors; and tibialis (TIB) is an ankle flexor. Total muscle activation here is calculated as the time integral of the linear envelope of the EMG curve (more details in the Experimental Section).

Muscle activations for all three walking conditions on Earth and Moon were measured. The Passive and Active conditions were then normalized by the muscle activations measured under the Earth‐None condition as baseline for comparison (see Figure 4A for Moon, and Figure S4 for Earth, Supporting Information). The EMG measurements of the seven analyzed muscles for a sample subject, showing activation patterns across all Earth and Moon walking conditions, are presented in Figure S5 (Supporting Information).

In Earth gravity (Figure S4, Supporting Information), we observed increased muscle activation of knee extensors and flexor (VM, VL, and BF) across all exosuit configurations. Specifically, the increase in muscle activation of VM, VL, and RF was found to be significantly related to the introduction of active resistance in the Earth‐Active condition (*n =* 6, two‐sided paired *t‐*test, *P =* 0.0146, 0.0041, 0.0195, respectively).

When applying active resistance (Moon‐Active condition), a further increase in muscle activation of the knee extensors and flexors was achieved: 20.3% (VM), 13.9% (VL), 39.8% (RF), 87.0% (BF), in comparison to the Earth‐None reference condition, as shown in Figure 4A. Activation of the ankle muscles was similar to the Moon‐Passive condition (SOL increased; GM and TIB decreased). These findings indicate that the exosuit's can stimulate muscle activation patterns in hypogravity to levels comparable to those observed under Earth gravity conditions. Furthermore, no statistically significant difference was found in the activation of the knee flexor/extensor muscles between Earth‐None and Moon‐Active (*n =* 6, two‐sided paired *t‐*test, *P =* 0.4592, 0.6168, 0.3906, 0.2071, respectively), indicating that these key muscle groups are being suitably stimulated by the active resistance of the exosuit in low gravity.

It has to be noted that muscle activation patterns shown in Figure S5 (Supporting Information) indicate that not only is muscle activation increased as described above, but it is also increased at biomechanically relevant points in time. This is clearly visible for VM, VL, and RF on Earth, and for VM, VL, RF, and BF, in simulated Moon gravity.

Figure 4B,C illustrates the difference between the muscle activation during the Moon conditions (Moon‐None and Moon‐Active) relative to the Earth‐None condition, represented as the “improvement measure” term. For the Moon‐None case, a decrease in muscle activation in all knee and ankle extensors was observed due to the lower gravity of the Moon environment, while the muscle activation of knee flexor BF and ankle flexor TIB increased. These changes in muscle activation from the Earth‐None condition happened due to weight offloading of the simulated Moon gravity (Moon‐None). Comparing Moon‐None and Moon‐Active conditions shows the impact of the exosuit's resistance on locomotion in Moon gravity; increased muscle activation is observed in all knee extensors (+24.4% in VM, +19.8% in VL, and 46.9% in RF), the knee flexor (+35.2% in BF), and two of the ankle muscles (+6.9% in TIB and +31.7% in GM), while SOL activation decreases (‐6.1%). Note that the activity of the GM increased with applied resistance; however, it (as well the activation of SOL) was still lower than the Earth's level. This suggests that while the exosuit's resistance can enhance muscle activation in the targeted muscles around the knee, it may not completely restore Earth‐like muscle activation levels in muscles around other joints.

### 2.3 Spatiotemporal Characteristics of Gait

**Figure 5:**
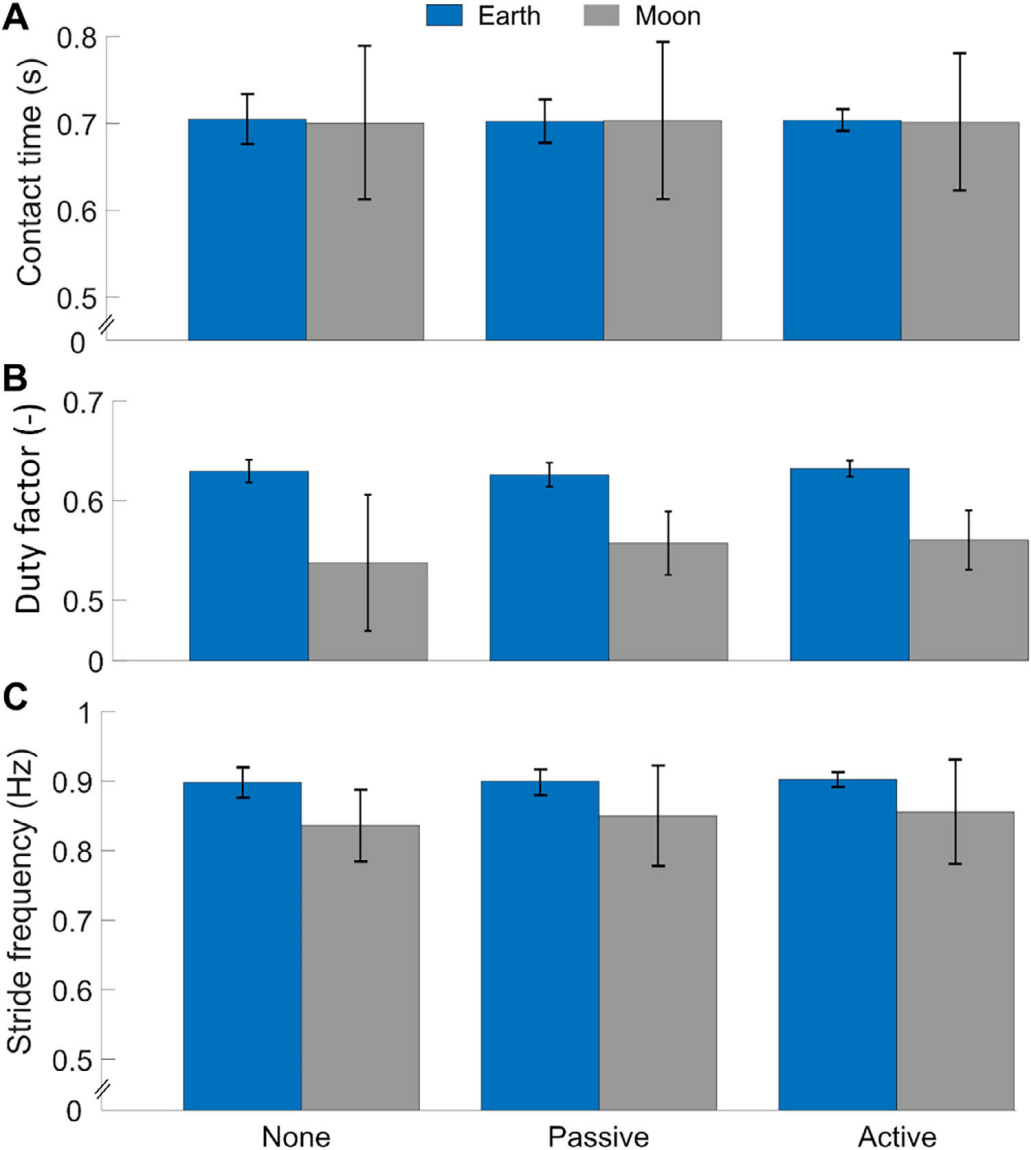
Spatiotemporal characteristics of participants’ gaits on Earth and Moon. A) Average contact time of the right foot, B) average duty factor, and C) average stride frequency of all three walking conditions on both Earth (blue bars) and Moon (gray bars). All parameters are shown for each testing configuration. Vertical bars indicate one standard deviation. No statistically significant changes in the measured foot contact time, duty factor, or stride frequency were found under any walking conditions, both on Earth and Moon. This suggests that the exosuit and the applied resistive forces did not significantly influence the subjects’ gaits.

To further investigate changes in gait when wearing the R‐HEXsuit, we measured foot contact time, duty factor (defined as the ratio between ground contact time and stride duration), and stride frequency of all three walking conditions in Earth and Moon gravity, as presented in **Figure** **5**. There was no statistically significant change in the measured foot contact time across all walking conditions, both on Earth and Moon. The average contact time on Earth was 0.700 ± 0.021 s, while that on Moon was 0.692 ± 0.085 s. Additionally, no statistically significant differences were detected in duty factor, both on Earth and Moon. The average duty factor on Earth was 63% ± 1%, while in simulated Moon gravity it was 55.4% ± 4.2%. Finally, no significant differences were found in the stride frequency across all three conditions and both gravities. Average stride frequency on Earth was 0.900 ± 0.015 Hz, and that on Moon was 0.807± 0.065 Hz. These findings indicate that the exosuit and the applied resistive forces did not significantly influence subjects’ gaits.

### 2.4 Resistive Forces Delivered by the R‐HEXsuit

To provide a clear understanding of the system's performance, we also analyzed the resistive forces applied through the actuators. In all Active tests, a proportional‐integral (PI) force controller was implemented to deliver resistance to the user's leg through the front and back BAM actuators. The controller received force inputs measured from the load cells mounted in line with the BAMs, calculated force errors from the resistive force demand and generated output pressure signals to control the pneumatic regulators to supply the BAMs during the targeted resistance period, shown in Figure 2B (more information can be found in the section “Control Strategy”).

**Figure 6:**
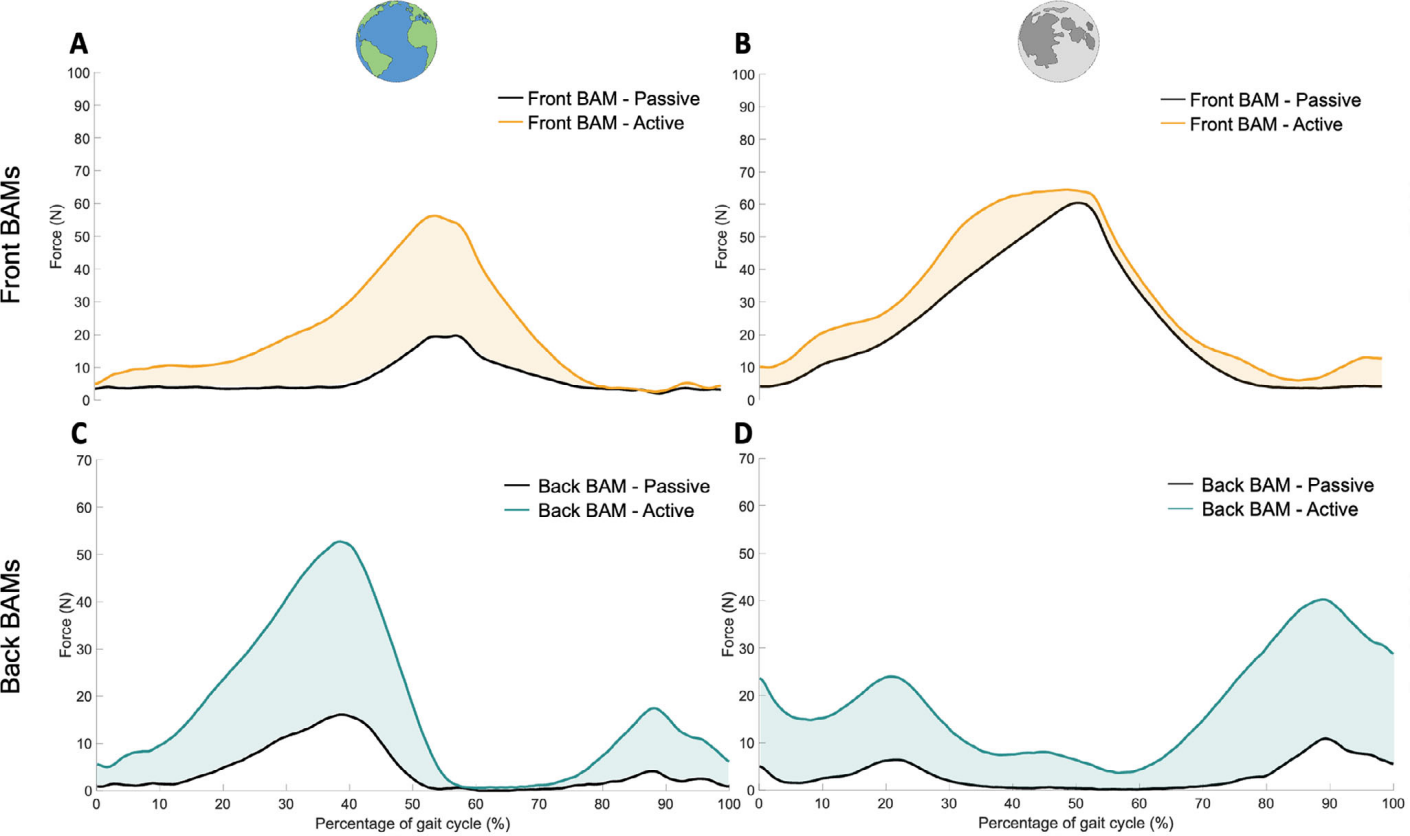
Average measured forces during walking on Earth A,C) and Moon B,D) gravity between Passive and Active conditions. Data are presented for one example subject for clarity, averaged across 20 strides where the 0% of the gait cycle was determined by the detection of the heel strike. Solid black lines present forces measured by the load cells during walking under Earth and Moon‐Passive; yellow and green solid lines represent measured forces during Earth and Moon‐Active conditions of the front and back BAMs, respectively. Orange and green shaded areas indicate increase in forces delivered from the front BAMs and back BAMs, compared to the Passive conditions.

**Figure** **6** illustrates the mean measured forces of the front and back BAMs during a gait cycle of 20 strides for a representative subject. For the powered resistance conditions (Earth‐Active and Moon‐Active), the resistive force demand was set to a ramp profile, starting from 0 N and linearly increasing to a maximum force of 60 N for the targeted resistance period shown in Figure S6 (Supporting Information). As a result, the actuation of the front BAMs in both Earth‐Active and Moon‐Active conditions (Figure 6A,B) led to an increase in the measured forces to the approximate peak of 60N as targeted within the resistance period. The peak measured forces of the back BAMs in Earth‐Active and Moon‐Active (shown in Figure 6C,D) were 52.68N and 40.17N, respectively. These lower forces than the target 60N resistance and observed change in the force profiles can be attributed to the slight change of gait characteristics due to the different gravity environments. In particular, altered knee flexion‐extension dynamics in hypogravity, such as reduced angular velocity or more extended postures, may limit the ability of the back BAMs to tension optimally within the controller‐defined resistance window. Previous studies^[^ ^57^ ^]^ observed a reduction in knee joint range of motion and a trend toward more extended postures under simulated low‐gravity conditions, along with minimal variation in knee phase space dynamics during swing, supporting this interpretation. This would reduce effective force build‐up before the actuator is unloaded. Additionally, since the same PI controller was used across all conditions, the observed reduction in performance suggests that controller timing may be suboptimal under Moon‐like gait kinematics.

### 2.5 Comfort Evaluation of the R‐HEXsuit

**Figure 7:**
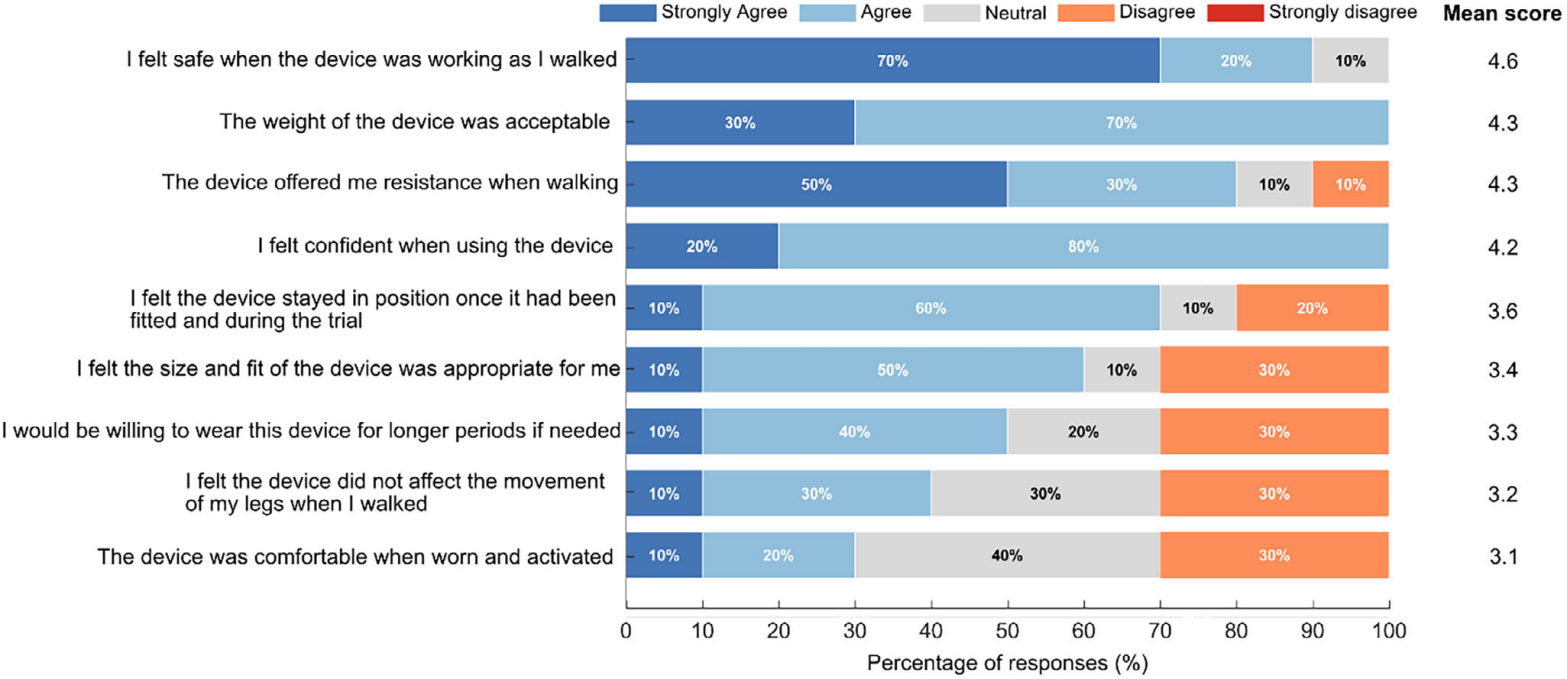
Distribution of responses to the R‐HEXsuit comfort questionnaire (*n* = 10) across five Likert categories. Participants completed three 4‐min treadmill walking bouts (no suit, suit powered off, suit powered on) under Earth gravity and then rated their agreement with comfort‐ and usability‐related statements on a 5‐point Likert scale.

A comfort evaluation was conducted under Earth gravity conditions, where participants (*n* = 10; 8 men and 2 women) walked on a treadmill for three 4‐min bouts: without the suit, with the suit worn but powered off, and with the suit powered on. Participants then completed a postevaluation comfort questionnaire using a 5‐point Likert scale (1 = Strongly disagree, 5 = Strongly agree) covering perceived safety, resistance, comfort, fit, weight, and willingness for prolonged use of the R‐HEXsuit (see the Supporting Information: Comfort evaluation questionnaire). The comfort evaluation was conducted under Earth gravity conditions. Responses indicated high perceived safety (mean ± SD, 4.6 ± 0.70) and confidence when using the device (4.2 ± 0.42), with the device weight rated as acceptable (4.3 ± 0.48). Perceived resistance during walking was also high (4.2 ± 1.03). Lower scores were observed for comfort when activated (3.1 ± 0.99) and for perceived absence of movement interference (3.2 ± 1.03). The stacked distribution of responses (**Figure** **7**) highlights that while the majority of participants agreed with positive statements, a subset reported neutral or negative ratings in comfort‐related items. The main source of discomfort, particularly reflected in responses to the statement “The device was comfortable when worn and activated,” arose from the knee straps slightly scratching the skin. This feature will be improved in subsequent versions of the suit. Willingness to wear the device for extended periods scored moderately (3.3 ± 1.06), reinforcing the need for these refinements in future iterations.

## 3 Discussion

In this work, we introduced the R‐HEXsuit, a first‐generation soft resistive hypogravity exosuit, designed to increase muscular loading and metabolic demands in the short term, with the long‐term objective of counteracting the musculoskeletal degeneration of exposure to hypogravity.

One key finding is the important role of active resistance in achieving meaningful physiological outcomes. In the simulated Moon gravity, the introduction of active resistance (Moon‐Active) led to a 29.3% increase in metabolic cost compared to not wearing the suit (Moon‐None) and a 7.7% increase compared to passive resistance (Moon‐Passive). The active exosuit restored metabolic expenditure on the Moon (Moon‐Active) to 1.9% above that of walking on Earth without wearing the suit (Earth‐None). Furthermore, activation of key muscle groups, such as the knee extensors and flexors, increased, ranging from 13.9% (VL) and 87% (BF), as shown in Figure 4A. These findings suggest that in simulated Moon gravity, the application of active resistance can achieve a meaningful increase in both metabolic expenditure and muscle activation, comparable to walking at Earth‐like levels. In contrast, passive resistance alone (Moon‐Passive) was insufficient to stimulate the desired levels of metabolic demand (‐5.3% compared to Earth‐None, Table S1.3, Supporting Information). The only exception was the biceps femoris (BF), where Moon‐Passive produced a 64.9% increase in muscle activity compared to Earth walking (Earth‐None).

On Earth, the R‐HEXsuit produced a 20% increase in metabolic cost in the Earth‐Active condition compared to no exosuit (Earth‐None), but only a modest 1.6% increase compared to passive resistance (Earth‐Passive). This suggests that a significant portion of the effective resistance in Earth gravity comes from the passive properties of the suit. However, active resistance applied in the Earth‐Active configuration led to a statistically significant increase in muscle activation of the vastus medialis (VM), vastus lateralis (VL), and rectus femoris (RF), compared to walking on Earth without the suit (Figure S4, Supporting Information). This is particularly evident in the timing and magnitude of muscle activation (Figure S5, Supporting Information), where active resistance resulted in higher peak activations at biomechanically relevant points. These findings highlight the critical role of active resistance in effectively stimulating the targeted muscles, both in simulated Moon gravity and Earth gravity.

Compared to existing designs, the R‐HEXsuit is the first prototype of a purpose‐designed resistive exosuit tested in both Earth and simulated Moon gravity. Recent reviews and experimental studies have explored resistive strategies in wearable robotics and clothing, including elastic textile‐based garments and soft exosuits for rehabilitation and upper‐body strength training.^[^ ^58^, ^59^, ^60^ ^]^ However, these devices are not directly comparable to the present work, as they address narrowly defined therapeutic or training goals that differ substantially from our intended application. Since there are no comparable resistive exosuits, Table S2 (Supporting Information) provides a summary of studies investigating the impact of assistive wearable exoskeletons on metabolic cost improvements. The reported reductions in metabolic cost range from 6% to 13%, with variability likely due to differences in exoskeleton design, targeted joints, and experimental protocols. Although some of these exoskeletons increase the metabolic cost of walking,^[^ ^29^, ^61^, ^62^, ^63^ ^]^ this is attributed to suboptimal assistance, or the added metabolic penalties imposed by the weight of the exoskeleton itself. On the other hand, the R‐HEXsuit is intentionally designed to increase the metabolic cost of walking as it imposes targeted resistance (active or passive) to the movement of the knee joint. In simulated low gravity, the active resistance of the suit increased the metabolic cost of walking by 29.3% when compared to walking without the suit (Table S1, Supporting Information).

We also compared the R‐HEXsuit with existing equipment that has also been designed to add resistance to the astronauts’ bodies to preserve musculoskeletal health (predominantly muscle mass and strength). The main devices used are the iRED (interim Resistive Exercise Device, now discontinued), the ARED (Advanced Resistive Exercise Device), and NMES (neuromuscular electrical stimulation). iRED and ARED enable resistive training through exercises, such as squats, deadlifts, and heel raises,^[^ ^64^ ^]^ whereas NMES is primarily used to maintain muscle mass (rather than strength) during static sessions lasting 30–60 min.^[^ ^65^ ^]^ **Table** **1** summarizes the modes of operation, roles, and typical use of each device, and introduces the R‐HEXsuit as a novel, wearable countermeasure designed to complement and integrate with existing approaches.

**Table 1:** Comparison of resistive and neuromuscular countermeasures relevant to spaceflight. The table outlines the modes of loading, typical loads or forces, functional roles, and typical uses of existing countermeasures (iRED, ARED, and NMES), and offers a comparison with the R‐HEXsuit.

Device	Mode of loading	Typical load/force	Role	Typical use
**iRED** (interim Resistive Exercise Device)^[^ ^64^ ^]^	Vacuum cylinder resistance	≤70–90 kg in practice; max ≈136 kg	Early resistive exercise device; limited effectiveness	Squats, deadlifts, heel raises performed daily; loading often capped by hardware limits
**ARED** (Advanced Resistive Exercise Device)^[^ ^64^ ^]^	Barbell‐like resistive exercise	Squats: 70–270 kg; Deadlifts: 90–270 kg; Heel raises: 110–270 kg	High‐load strength and bone preservation	2× daily sessions (≈1–1.5 h); structured resistive training similar to Earth‐based barbell workouts
**NMES** (Neuromuscular Electrical Stimulation)^[^ ^65^ ^]^	Electrical stimulation of muscles	≈20% MVC (maximum voluntary contraction) force (intensity‐limited by tolerance)	Passive contractions, prevents atrophy in key muscles	Applied to knee extensors/plantar flexors during sedentary periods; 30–60 min per session, ideally 2 times per day
**R‐HEXsuit** (Resistive Hypogravity Exosuit; this work)	Pneumatic actuators applied during gait	≈60 N per actuator (≈6 kg per leg)	Submaximal, continuous resistance during locomotion	Worn during walking/treadmill tasks, routine activities in planetary missions, and as a complementary countermeasure to restore Earth‐like energy cost and muscle activation in hypogravity

A further advantage of the R‐HEXsuit lies in its ability to deliver resistance at biomechanically relevant points without impairing the user's mobility and gait, as evidenced by the consistent stride frequency, contact times, and duty factor recorded (Figure 5; and Figure S5, Supporting Information). This is an appealing feature both for incorporation into future space suits, and for potential Earth applications. In space, the vision is to integrate the R‐HEXsuit (e.g., as an undergarment) seamlessly into mission activities, especially during daily tasks. Consequently, comfort emerges as a primary concern, with the R‐HEXsuit offering distinct advantages over conventional countermeasures that impose restrictions on the user's range of motion or prove uncomfortable for continuous wear.^[^ ^66^, ^67^ ^]^ This feature is highly desirable when considering integration with current and future space suits, such as the Extravehicular Mobility Unit (EMU).^[^ ^68^ ^]^ However, implementing the R‐HEXsuit within EMUs will require overcoming several practical challenges. A key consideration is ensuring adequate clearance between the exosuit and the EMU interior, as current suits already impose considerable mobility constraints due to their bulk and rigidity. Additionally, integrating pneumatic supply systems, such as valves, compact air tanks, or miniature pumps, and associated control electronics into the already densely packed EMU architecture poses a nontrivial systems engineering challenge. That said, because EMUs effectively function as self‐contained spacecraft equipped with Environmental Control and Life Support Systems (ECLSS), the incorporation of compact pneumatic elements could potentially leverage existing infrastructure. This might make such integration more feasible compared to standalone hardware additions. Addressing these integration aspects early in the design process, especially through analog field tests in simulated planetary suits, will be critical to realize a deployable version of the R‐HEXsuit for space applications.

Future studies will investigate higher resistive forces, particularly for Earth‐based applications. Scenarios such as rehabilitation and resistive training augmentation often require greater loads than those encountered when walking, prompting modifications to the suit to enhance force transmission from the actuators. This will include the development of novel soft anchoring devices to optimize performance. Improving the force transmission of our exosuit requires addressing the compliance of its soft robotic actuators. The BAM actuators used in this study are soft, textile‐based pneumatic artificial muscles that generate force and stiffness by pressurising air within them. One foreseeable approach to address this will focus on refining the anchoring designs and actuator performance to achieve more effective and reliable force transmission.

Future studies will also include supplementary biomechanical measurement sensors, embedding them directly into the device to create a fully sensorized, untethered system. With the added sensing, the suit could also include functionalities to augment resistive exercise in the habitat, including fundamental resistive exercise movements, such as squats, deadlifts, and jumping.

The current version of the R‐HEXsuit only targets the knee joint, with effects on other joints (hip and ankle) still unexplored. Future studies will involve extending the exosuit to encompass additional joints, such as the hip and ankle, to provide comprehensive resistance to all joints involved in walking. Targeting the plantar flexors (e.g., gastrocnemius and soleus), which are highly affected by hypogravity,^[^ ^6^, ^69^ ^]^ could enhance the suit's ability to preserve lower limb strength. However, this introduces design and control challenges, requiring additional effort in device development. The lower limb joints are biomechanically coupled, so applying resistance at multiple joints affects overall gait dynamics. Coordinating multiple soft actuators across joints will require advanced sensing, real‐time gait phase detection, and adaptive control strategies to maintain natural movement patterns. An important next step will be to evaluate the long‐term effects of wearing the exosuit on overall musculoskeletal health. Long‐term effects, such as user fatigue, neuromuscular adaptation, or possible changes in gait strategy remain unexplored. Future studies should assess the cumulative impact of daily use over extended durations. In particular, evaluating the R‐HEXsuit in a ground‐based analog, such as a long‐duration bed rest campaign, would help determine whether regular walking with resistive loading could serve as a viable standalone countermeasure to preserve musculoskeletal health in hypogravity. This research will include a larger sample size to enhance the study's statistical power and a more diverse range of participants, accounting for variations in age, fitness levels, and gender. Ideally, the exosuit would also be tested on actual astronauts; however, the candidate pool for such studies would be inherently limited.

Future versions of the R‐HEXsuit could also include assistive functionalities, to aid the astronauts during long extravehicular activities, and during habitat construction. This could be achieved by exploring alternative control and actuation strategies, leading to a multifunctional device that could be adopted across all stages of the mission. The R‐HEXsuit could be adapted for running, but this would require a significant overhaul of the control strategy due to (in the case of running) the shorter stance phase, flight phase, and faster joint dynamics. Particularly, the R‐HEXsuit was programmed to provide resistance during the stance phase of walking. Integrating inertial sensors (IMUs) would be key for real‐time gait detection and timely actuation for nonwalking gaits. However, the use of IMUs in space remains challenging, as their performance can be affected by altered gravitational conditions. To address this, recent advances in wearable sensing have demonstrated that distributed fiber‐based strain sensors embedded in garments can achieve reliable joint angle reconstruction without relying on gravity‐dependent reference frames.^[^ ^70^ ^]^ Reviews of functional fibers and fabrics have further highlighted how textile‐integrated sensing offers mechanically adaptable, skin‐conformal solutions for motion monitoring and human–robot interaction.^[^ ^71^ ^]^ In parallel, developments in wearable and stretchable strain sensors based on resistive, capacitive, and optical mechanisms have shown robust performance for human motion detection and soft robotics applications.^[^ ^72^ ^]^ Together, these approaches are not dependent on gravitational cues and therefore hold promise for space applications where IMUs may be less reliable. Moreover, running is unlikely to be relevant in hypogravity, where astronauts tend to adopt energy‐efficient gaits, such as walking. Prior studies^[^ ^43^ ^]^ confirm that walking requires less metabolic energy, followed by skipping and running in lunar conditions.

Future versions of the R‐HEXsuit could also incorporate real‐time EMG processing to dynamically adjust resistance based on the user's muscle activity. This would allow the exosuit to personalize loading based on fatigue, asymmetry, or reduced engagement, optimizing the training stimulus while avoiding overuse. AI‐driven control strategies using EMG inputs represent a promising direction for enhancing adaptability during long‐duration missions or rehabilitation scenarios.

This work demonstrates the potential for the R‐HEXsuit to become a core component of long‐term spaceflight, acting as a comfortable and effective countermeasure to musculoskeletal degradation. Its versatility, efficacy, and compatibility to the human body make it suitable for use in conjunction with exercise equipment and as a daily garment, allowing astronauts to experience Earth‐like loads during routine tasks. The R‐HEXsuit lays the foundation for a step‐change in maintaining astronaut musculoskeletal health during extended space missions in low gravity, potentially making a crucial contribution to the success of future manned space exploration.

## 4 Experimental Section

### Soft Exosuit Design

The soft R‐HEXsuit exosuit was designed to resist knee extension and flexion during walking (Figure 1D). The textile components of the suit include a neoprene bilateral hip‐to‐thigh brace with Velcro attachments for easy fitting, customized foot attachments, and the soft bubble artificial muscles connecting the front region of the hip brace to the foot attachments.

The R‐HEXsuit is adjustable to fit different participants using adjustable straps on two locations of the suit. Cam buckles (Fasty, Sweden) were sewn into the textile components of the suit and webbing straps were used to adjust the hip‐to‐thigh length between the hip part and the thigh part, and the BAM length between the thigh part and the front or back of the foot attachments. This approach allows for easy and controlled positioning of the BAMs along the lower limbs. Additional knee braces were fabricated to place over the knee to secure and maintain the position of the front BAMs around the patella.

### BAMs Fabrication and Design

The bubble artificial muscle is a linear contractile pneumatic actuator made from a thin folded tube by an addition of constraining rings with smaller radius than the tube, placed along the tube at equal spacing. When inflated, the folded tube between the rings unfolds and forms an elliptical shape as its maximum inflated shape, achieving a linear contraction of up to 43.1%.^[^ ^42^ ^]^ In this study, each BAM was fabricated using TPU‐coated (thermoplastic polyurethane) Nylon textile (Riverseal 70 LW, Rivertex, UK), a commercial clothing material. The use of nylon textile makes the actuators resistant to high pressures and facilitates their ready integration into clothing using textile assembly methods.

A textile tube (the precursor tube for BAM fabrication) was created by heat sealing two TPU‐coated textile sheets together, using a CNC machine (Ooznest Limited, UK) with a custom soldering iron as an end effector (T0052918099N, Weller Tools, Germany). A pneumatic fitting was incorporated into each actuator to enable airtight connections. Subsequently, a set of constraining rings was pushed along the tube to form the contractile units of the actuator. The BAM performance is influenced by contractile unit length, ring radius, and material thickness, as described in ref. [42]. Therefore, the BAMs used in this study were fabricated to have a maximum expanded diameter of 40 mm, with a contractile unit length of 54 mm and a ring radius of 3.5 mm. Pairs of BAMs were connected in parallel to form the front BAMs, containing five contractile units (total length 270 mm), and the back BAMs, containing six contractile units (total length 432 mm), for each leg and configured as shown in Figure 1D. The front BAMs were attached to the thigh brace at mid‐thigh at the top end and above the ankle attachment at the bottom end. The front BAMs were also secured through the knee brace to maintain their position at the front of the knee. The back BAMs were attached to the thigh brace at one third of the thigh length from the hip joint at the top end and wrapped around the thigh to connect to the back of the ankle attachment. A detailed diagram of the attachment points in relation to the user's bone anatomy is shown in Figure S1 (Supporting Information).

### Sensing and Control

The suit was equipped with four miniature load cells (S Type Load Cell DYLY‐106 30 kg, CALT, USA), to measure the force exerted in line with the front and back BAMs on both legs. The load cell signals were amplified using load cell amplifiers (BRT RW‐ST01A, Brightwin Electronics, USA). The pressures inside the actuators were measured using miniature absolute pressure sensors, one for each actuator (SSC‐D‐AN‐N‐030PD‐AA5, Honeywell). Two force sensitive resistors (FSR, RP‐S40‐ST, Hilitand, USA) were attached to the bottom of a shoe sole at the heel and toe to detect heel strike and toe off events, respectively. These distinguished gait phases and are used to generate control signals for the BAMs and to calculate stride frequency and contact time.

Pressurized air was supplied to the BAMs using four digital pressure regulators (ITV2050‐31F3N, SMC, UK), one for each pair of BAMs. The pressure regulators were controlled by an Arduino Mega board (Arduino, Italy), running a PI (proportional‐integral) controller. Data from the exosuit sensors were acquired by a data acquisition device (USB‐6211, National Instruments, USA) at a rate of 1000 samples s^−1^.

### Fitting Procedure of the Exosuit

Under the Earth‐Passive condition (walking with the passive exosuit on Earth), the artificial muscles were unpressurized, and the maximum passive tension of the actuators was adjusted and limited at a peak of 20 N for all subjects, for both front and back BAMs. This one‐time tension adjustment was done at the beginning of the Earth‐Passive condition to prevent slack in the actuators during further tests. No further adjustments were made on the user's leg for any walking conditions on both Earth and Moon.

### Control Strategy

The force demand to deliver resistance to a lower limb was set as a ramp profile starting from 0 N at the beginning of the resistance period and ending with a maximum force of 60 N (three times the passive tension of 20 N set in Earth‐Passive) at the end of the resistance period. This value was chosen after tests conducted in the lab showed that a greater force than this was uncomfortable to the user. The resistance period of the back BAMs for knee extension was 10%–45% of gait cycle, and that of the front BAMs for knee flexion is 40%–60% of gait cycle, as illustrated in Figure 2.

The proportional‐integral (PI) controller was designed to take input force measurements from the load cells, calculate force error with respect to the force demand and generate an output in the form of a pressure signal to the regulators which supply pressurized air to BAMs. The controller gains were tuned using the Ziegler–Nichols method, following a similar procedure as described,^[^ ^43^ ^]^ where *K*_P_ and *K*_I_ are the proportional and integral gains, respectively. The tuned values were *K*_P_ = 10.0 and *K*_I_ = 0.01, applied universally to both front and back actuators across all conditions.

The controlled pressure was applied at the selected gait phases as described above. The actuation period of the BAMs was calculated at every gait cycle based on the gait cycle period 𝑇, which was continuously updated by averaging the time between two sequential heel strikes over the three last steps. The heel strike events at the beginning (0%) and end (100%) of the gait cycle and the toe‐off event (approximate at 55% of the gait cycle, varying sightly for each individual) were detected using the insole force sensors and converted to a percentage of gait period T, using Equation (1). These values were used to determine the start and end time of actuation for each pair of BAMs as follows

(1) $$TO_{\%\;}=TO_{\mathrm{time}}/T$$

(2) $$\mathrm{Front}\;\mathrm{BA}M_{\mathrm{Start}\%}=TO_{\%}-10\%$$

(3) $$\mathrm{Front}\;\mathrm{BA}M_{\mathrm{End}\%}=TO_{\%}+5\%$$

(4) $$\mathrm{Back}\;\mathrm{BA}M_{\mathrm{Start}\%}=10\%$$

(5) $$\mathrm{Back}\;\mathrm{BA}M_{\mathrm{End}\%}=TO_{\%}-10\%$$

where TO_time_ is the timestamp of toe‐off events and TO_%_ is the percentage of the toe‐off event within the gait cycle. Front BAM_Start%_ and Back BAM_Start%_ are the start percentages of actuation of front and back BAMs, respectively, while Front BAM_End%_ and Back BAM_End%_ represent their respective end percentages of actuation. These percentages were later converted to corresponding time durations in order to control the actuation of the BAMs.

To account for the inherent delays of the pressure regulators (typically 100 ms between change in the demand and change in the pressure outputs) and to ensure rapid response of the actuators, the pressure regulators were set to deliver a pressure of 50 kPa for a duration equivalent to 15% of the walking period 𝑇 prior to the main actuation of each BAM.

### Human Study

Six healthy adult participants were recruited (*n* = 6; four men and two women; averaged ± SD age of 30.8 ± 6.7 years; mass 64.4 ± 7.5 kg, height 1.76 ± 0.03 m), physically active. Ethical approval for the study was obtained from the University of Bristol Research Ethics Committee (application 17 932), and all participants provided written consent after receiving comprehensive information about the study's objectives and potential risks. Prior to the testing sessions, participants underwent a thorough familiarization process with both the system and the testing facility to minimize any unexpected outcomes.

### Experiment Design and Protocol

The effect of the suit on the metabolic cost and electromyographic (EMG) activation patterns was tested for two different levels of gravity (Earth at 1 g, and Moon at 0.16 g), at one speed of walking (1.1 m s^−1^) for 4 min to allow the oxygen consumption to reach steady state (see “Metabolic rate measurements” for more details). Before starting the experiments, each participant was asked to stand quietly, with all sensors mounted on the body but not wearing the exosuit, to measure resting oxygen consumption. Each participant undertook all Earth gravity tests before being fitted in the gravity compensation system and undertaking all Moon gravity tests.

First, in Earth gravity, the participant was asked to walk on the treadmill with no exosuit on (Earth‐None). Subsequently, the exosuit was put on, and the tension of the BAMs was adjusted to a maximum of 20 N for each participant. This was done by performing three 10 s periods of walking, between which the load cell outputs were checked, and adjustments made to the position of the BAMs to obtain the desired tension. The subject was then asked to walk with no resistance provided (exosuit worn but passive resistance provided, Earth‐Passive). The same walking task was repeated while providing the resistance profile described above. When resistance was applied (Earth‐Active), back BAMs resisted knee extension (10%–45% of the gait cycle), while front BAMs resisted knee flexion (40%–60% of the gait cycle).

After about 20 min of rest, the tasks were repeated, excluding familiarization and quiet standing, under simulated Moon gravity.

### Facility—Hypogravity Analog

The L.O.O.P. (Locomotion On Other Planets) facility (Department of Pathophysiology and Transplantation, University of Milan; Milan, Italy), an ESA (European Space Agency) ground‐based facility, allows the simulation of low gravity by means of body weight suspension. The facility is located in a cavaedium (vent shaft). This provides a narrow (3 m × 3 m) and tall (17 m) space at the top of which the pully suspension system is mounted which support participants over a floor‐mounted motorized treadmill (Bertec). The suspension device is formed by two bungee jumping rubber bands (Exploring Outdoor srl, Italy), with a resting length of 4 m and a stiffness of 92.7 N m^−1^, and is linked in‐series by an inextensible short cable (Gottifredi & Maffioli, Italy, Dyneema SK78, diameter 4 mm, length 1.2 m) and extended through the adjustable top pulley. One end of the rubber band is fixed to the wall and the other end is connected to a participant's harness. The mobile pulley can be lifted or lowered by means of a suspension cable connected to a motorized winch (2.20 kW, Officine Iori SRL, Italy) to unload the body by the desired vertical force checked by means of a scale and a force transducer (REP Transducers, TS 300 kg, Italy) in‐series with the suspension cable. The experimental set up and a diagram of the facility can be found.^[^ ^44^, ^45^ ^]^

While this apparatus effectively simulates low‐gravity conditions by applying a constant vertical force to the body's center of mass, it is important to note that the pendulum‐like dynamics of swinging limbs remain influenced by Earth's gravity. However, this has a negligible effect on oxygen consumption and, consequently, on metabolic cost.

### Metabolic Rate Measurements

Respiratory gases, pulmonary ventilation, oxygen consumption (V̇O_2_, mlO_2_ kg^−1^ min^−1^) and carbon dioxide production (and the resulting respiratory exchange ratio) were measured breath‐by‐breath by a portable metabolic system (K5, Cosmed, Italy). Each experimental session started with a 4‐min resting V̇O_2_ assessment, while participants were standing, after which participants started walking on the treadmill. Each walking data acquisition session lasted 4 min, which are sufficient to collect steady‐state V̇O_2_ data (as shown in ref. [45]).

The metabolic cost of walking, i.e., the metabolic energy needed to move 1 kg of body mass for 1 m (C, J kg^−1^ m^−1^),^[^ ^73^ ^]^ was calculated as

(6) $$C=\frac{\left(\dot{V}O_{2\mathrm{ss}}-\dot{V}O_{2\mathrm{rest}}\right)\mathrm{Eq}O_{2}}{v}$$

where V̇O_2ss_ and V̇O_2rest_ are the oxygen consumption during the last minute of walking and standing rest, respectively, *v* is the average walking speed (m s^−1^), and EqO_2_ is the number of Joules released from the oxidative combustion of 1 mL of oxygen at a given respiratory exchange ratio (based on the respiratory exchange ratio measured for each trial and participant). As expected, in all the conditions, walking was at submaximal intensity with a respiratory exchange ratio < 1. Participants were instructed to avoid strenuous exercise in the 24 h before and avoid caffeine or food for at least 3 h before the experiment.

### Electromyography (EMG)

Seven EMG sensors (Delsys Trigno system, USA) were placed on the major lower limb muscles involved in walking as specified in ref. [74]. The muscles targeted were the knee extensors vastus lateralis (VL), vastus medialis (VM), and rectus femoris (RF), the knee flexor, bicep femoris (BF), and the ankle muscles, soleus (SOL), tibialis anterior (TIB), and gastrocnemius medialis (GM). Skin was shaved and rubbed with an alcohol wipe to reduce electrode‐skin impedance and to achieve a better fixation of the electrodes. Alcohol was allowed to vaporize before sEMG sensors were placed.^[^ ^74^ ^]^ During the last 60 s of each walking condition, EMG data were acquired in units of millivolts (mV) at a sample frequency of 2000 Hz and synchronized with triggers sent from the main data acquisition card (DAQ, National Instrument, USA).

The Delsys hardware automatically bandpass filtered all EMG signals (20–450 Hz). The raw EMG signals were high‐pass filtered, full‐wave rectified and then low‐pass filtered using a 4th order IIR Butterworth zero‐phase filter with cut‐off frequencies 50 Hz (high‐pass) and 20 Hz (low‐pass for creating the linear envelope of the signal) as described.^[^ ^75^ ^]^ The EMG signals (in mV) were sectioned by each stride (heel strike to heel strike period) and were time‐normalized to 201 points for each stride. The EMG signals for all strides were averaged, resulting in the average EMG signal during a gait cycle (shown in mV in Figure S5, Supporting Information).

Subsequent analyses were performed to obtain the total activation of each muscle. First, the time integral of the linear envelope of the averaged EMG signal was calculated (consistent with^[^ ^76^ ^]^) as an index of the muscles’ overall activity in each condition. This method of analysis can be physiologically interpreted as the time under tension of the muscle, which is an indicator of the intensity of the training stimulus and can be used for comparison between activities. Later, the time integral of each muscle and activity was normalized by the activation during Earth walking with no suit on (Earth‐None) in order to highlight relative changes between conditions.

Figure 4A presents the ratio of the muscle activation between different walking conditions (no unit); Figure 4B,C presents the difference of the muscle activation between different walking conditions, represented as “improvement measure” with the unit of mV.

### Statistical Analysis

Mean and standard deviation for metabolic cost and muscle activation were calculated for each condition, and for each subject. The effect of wearing the exosuit both active and passive by performing multiple comparisons was assessed. To this end, two‐sided paired *t‐*tests were performed with Holm–Šidák correction applied to account for multiple comparisons (significance level *α* = 0.05, Matlab). This technique was chosen as the standard technique used in other similar studies.^[^ ^29^, ^31^, ^77^ ^]^

## Conflict of Interest

The authors declare no conflict of interest.

## Author Contributions

E.P., R.S.D., and G.P. contributed equally to this work. Conceptualization: E.P., R.S.D., H.H., J.R.; Methodology: E.P., R.S.D., G.P., J.R.; Investigation: E.P., R.S.D., G.P., V.N., A.M.; Data analysis: E.P., R.S.D., G.P.; Supervision: R.S.D., H.H., J.R.; Funding acquisition: E.P., R.S.D., H.H., J.R.; Writing – original draft: E.P., R.S.D., G.P.; Writing – review and editing: E.P., R.S.D., G.P., H.H., J.R.

## Supporting information



Supporting Information

Supplemental Movie 1

Supplemental Movie 2

Supplemental Movie 3

Supplemental Movie 4

## Data Availability

Data for this study are available at the University of Bristol data repository, at https://doi.org/10.5523/bris.j4p2y997d312ll4kl4upuoas
