# Supplementary material for: A Resistive Soft Robotic Exosuit for Dynamic Body Loading in Hypogravity
Source: Adv Sci (Weinh). 2025 Sep 30;12(47):e06057. doi: 10.1002/advs.202506057 (PMC12713045; doi:10.1002/advs.202506057)
Supplement: Supplementary file 1 — Supporting Information [file ADVS-12-e06057-s003.pdf]

Supplementary Materials for

A Resistive Soft Robotic Exosuit for Dynamic Body Loading in Hypogravity

Emanuele Pulvirenti *et al.*

Corresponding author. Email: jonathan.rossiter@bristol.ac.uk

This PDF file includes:

Table S1 Percentage comparison of the metabolic expense between two groups of walking conditions.
Table S2 Overview of studies on metabolic cost impact using wearable exoskeletons.

Figure S1 Detail of attachment points of actuators with relation to the bone anatomy.
Figure S2 Diagram of a single leg to illustrate the muscles monitored during the study.
Figure S3 Average metabolic expense measurements on Earth for each testing condition.
Figure S4 Muscle activation of walking under Earth-Passive and Earth-Active conditions.
Figure S5 Average EMG measurements of the seven main muscles involved in walking.
Figure S6 Actuation profiles of the force demands set for back and front BAM actuators.
Comfort evaluation questionnaire. This includes the set of questions used in the R-HEXsuit comfort questionnaire.

Other Supplementary Materials files for this manuscript:

Movie S1 Actuation of the R-HEXsuit during different phases of the walking gait.
Movie S2 Demonstration of range of motion available during lower body movements while wearing the R-HEXsuit.
Movie S3 Demonstration of gaits in Moon gravity (0.16g) while wearing the R-HEXsuit.
Movie S4 Side-by-side comparison of walking gaits without wearing suit, applying passive resistance, and applying active resistance.

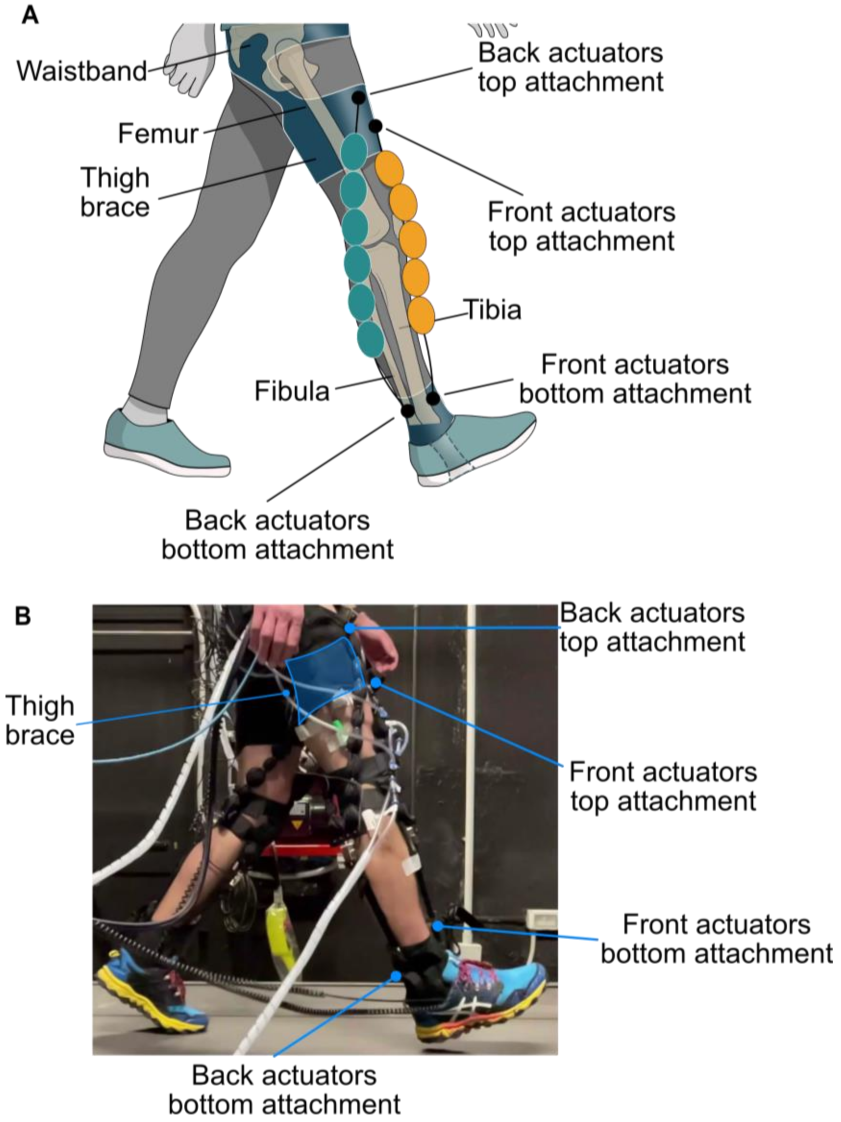

Figure S1. Detail of attachment points of actuators with relation to the bone anatomy of the user. (**A**) Schematic showing fixing points, and (**B**) Labelled photograph of exosuit in use. The top attachments of the front actuators were attached to the front of the thigh brace at mid-thigh (middle of the femur), and their bottom attachment were attached at the front of the ankle. The top attachments of the back actuators were attached to the back of the thigh brace at one third of the thigh length from the hip joint (one third of the femur) and wrapped around the lateral sides of the thigh to connect to the back of the ankle as their bottom attachment.

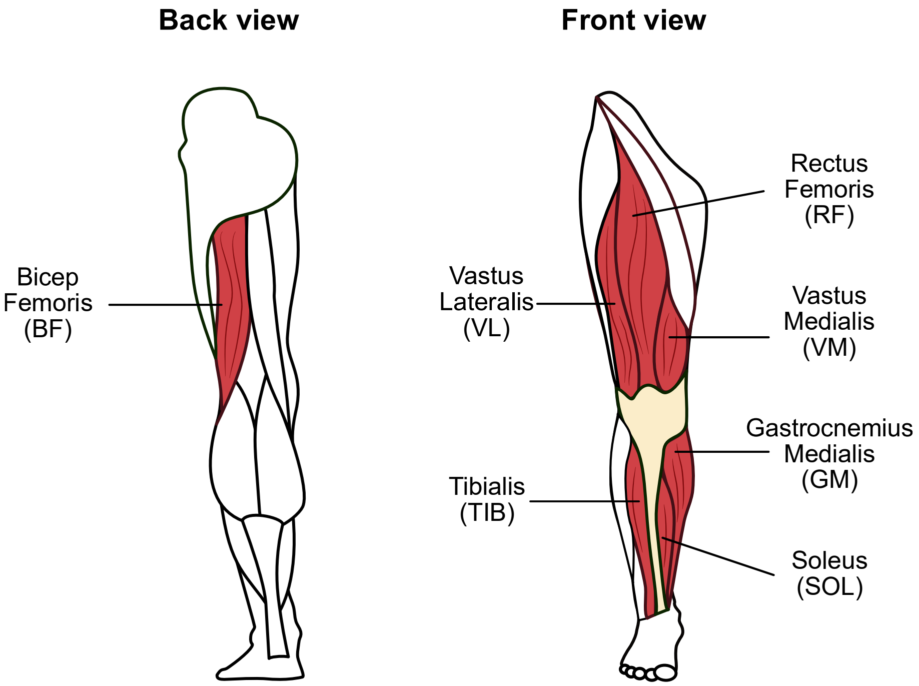

Figure S2. Diagram of a single leg to illustrate the muscles monitored during the study.

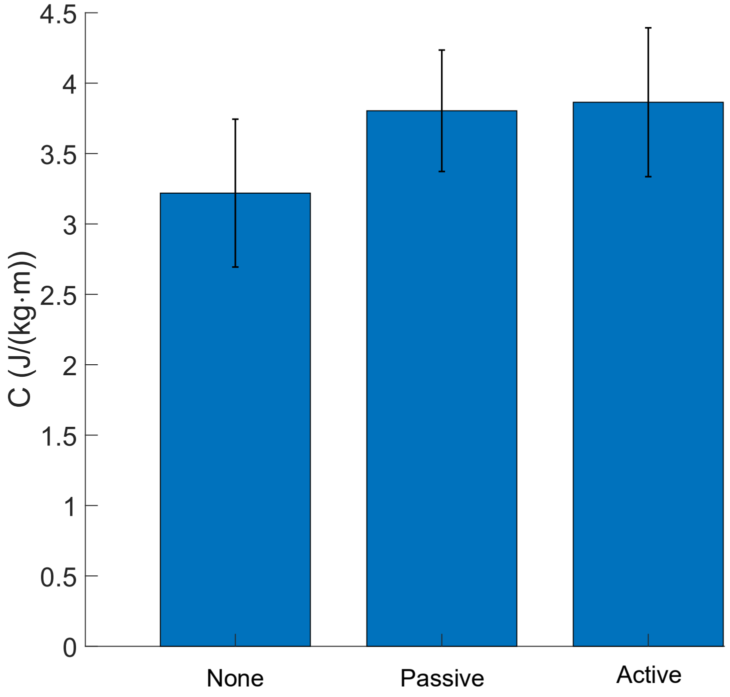

Figure S3. Average metabolic expense measurements on Earth for each testing condition. These include not wearing the exosuit (Earth-None), wearing the exosuit without activation (Earth-Passive), and applying resistance (Earth-Active).

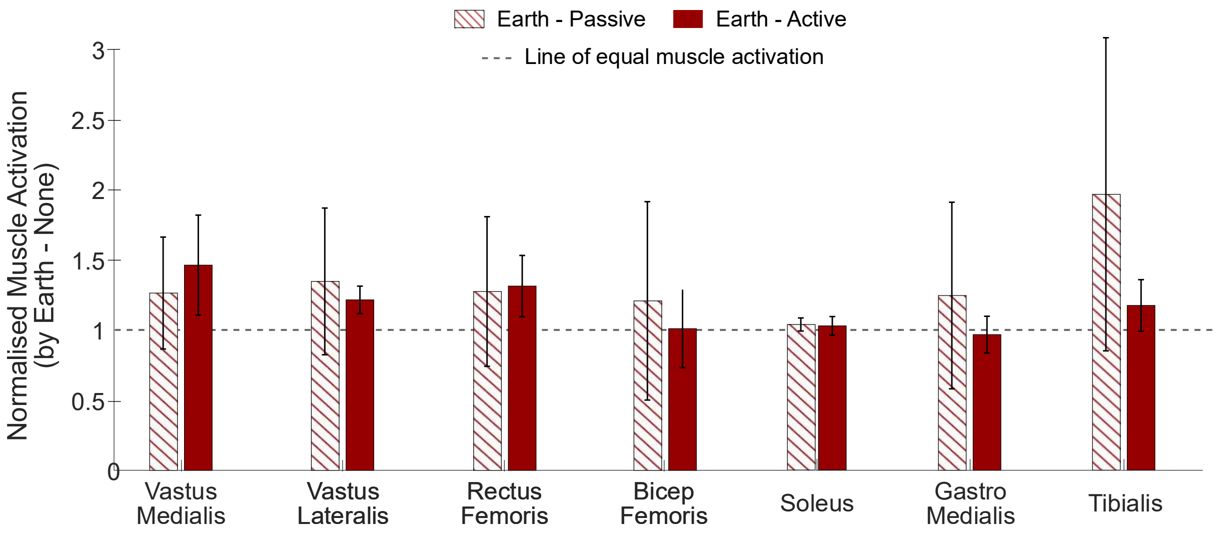

Figure S4. Muscle activation of walking under Earth-Passive and Earth-Active conditions. Both conditions are normalized by the Earth-None condition. The horizontal dashed line indicates equal muscle activation compared to the Earth-None condition. The measured muscles contain knee extensors (vastus lateralis, vastus lateralis and rectus femoris), knee flexor (bicep femoris), ankle extensors (soleus and gastro medialis) and ankle flexor (tibialis).

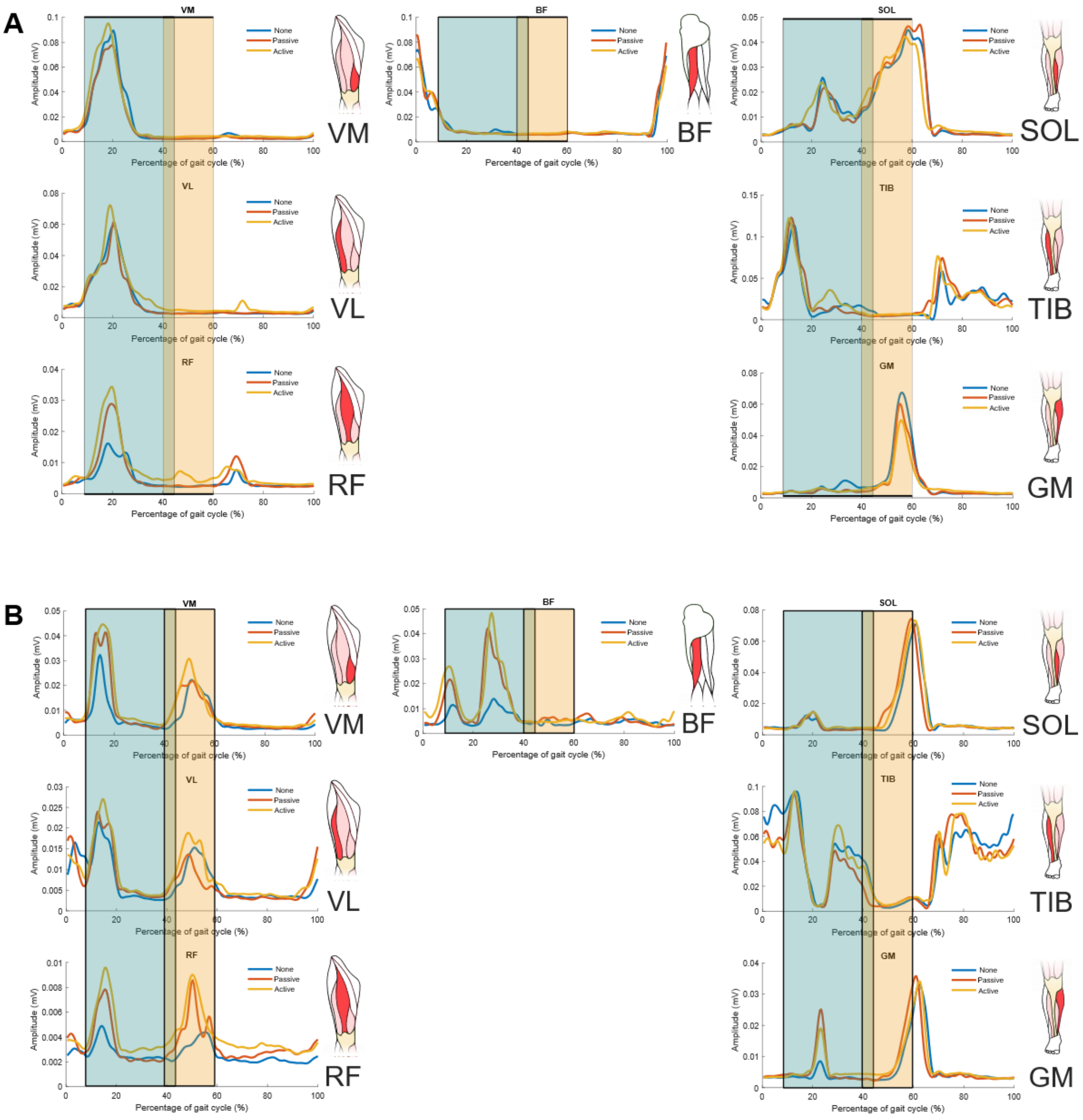

Figure S5. Average EMG time course of the seven main muscles involved in walking. The average EMG measurements through the gait cycle of each muscle for three walking conditions, including (**A**) Earth-None, Earth-Passive, and Earth-Active and (**B**) Moon-None, Moon-Passive, and Moon-Active of a single subject. Shaded green areas indicate the region of actuation of the back BAMs (actuated between 10% and 45% of the gait cycle), and shaded orange areas indicate actuation of the front BAMs (actuated between 40% and 60% of the gait cycle). Inset diagrams illustrate measured muscles, represented in red.

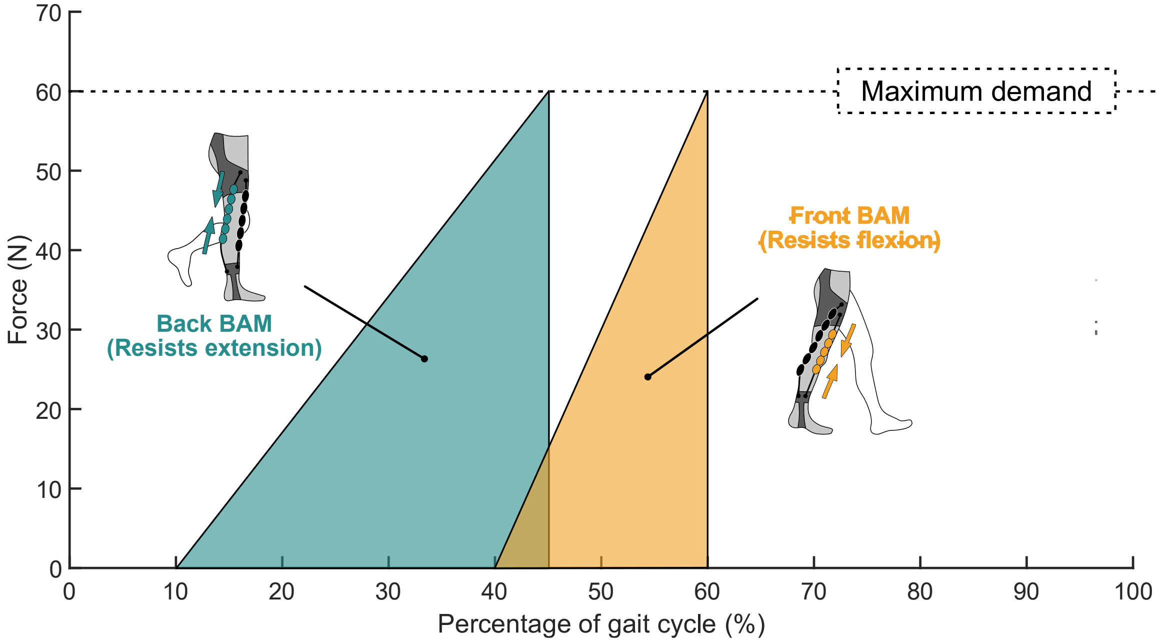

Figure S6. Actuation profiles of the force demands set for back and front BAM actuators. The force demands were designed as a ramp profile for the duration of the device actuation with the maximum force demand of 60 N. The Back BAMs were activated between 10% and 45% of a gait cycle (green profile); the Front BAMs were activated between 40% and 60% of the gait cycle (orange profile).

		Earth		
		None	Passive	Active
**Earth**	None	0.0	**18.2***	**20.1***
	Passive	-15.4	0.0	**1.6**
	Active	-16.7	-1.6	0.0

Table S1.1

		Moon		
		None	Passive	Active
**Moon**	None	0.0	**20.1***	**29.3***
	Passive	-16.8	0.0	**7.7***
	Active	-22.7	-7.1	0.0

Table S1.2

		Moon		
		None	Passive	Active
**Earth**	None	**-21.2**	**-5.3***	**1.9***
	Passive	**-33.3**	**-19.9**	**-13.7**
	Active	**-34.3**	**-21.1**	**-15.1**

Table S1.3

Table S1. Percentage comparison of the metabolic expense between two groups of walking conditions. The walking conditions include walking on Earth (None, Passive, and Active) and Moon (None, Passive, and Active). The numbers represent the percentages which were calculated from the change of the column value from the row value divided by the row value. S1.1 compares Earth to Earth conditions, S1.2 compares Moon to Moon conditions, S1.3 compares Moon to Earth conditions, and Asterisks indicate the values mentioned and used in the manuscript.

Ref. no	Mode	Type	Target joint(s)	Impact on metabolic cost	No. of subjects
[S1]	Assist	Rigid	Ankle	8 ± 3% reduction	7
[S2]	Assist	Rigid	Hip	Up to 6% reduction	10
[S3]	Assist	Soft	Hip	9.3% reduction	9
[S4]	Assist	Soft	Hip	Up to 13.05% reduction	7
[S5]	Assist	Soft	Ankle	12% reduction compared to normal walking; 21% reduction compared to exoskeleton deactivated	10
[S6]	Assist	Soft	Hip	11.52% reduction	6
[S7]	Assist	Soft	Ankle (Hemiparetic gait)	10 ± 3% reduction	9
**This work**	**Resist**	**Soft**	**Knee (Earth and hypogravity walking)**	**20% *increase* in Earth's gravity, 29.3% *increase* in simulated Moon gravity**	**6**

Table S2. Overview of studies on metabolic cost impact using wearable *assistive* exoskeletons. The table summarises the type of exoskeleton (rigid or soft), the mode of operation, the joint(s) targeted, the percentage impact on metabolic cost during use (compared to walking with no device), and the number of subjects involved in each study.

[S1] L. M. Mooney, E. J. Rouse, H. M. Herr, Autonomous exoskeleton reduces metabolic cost of human walking during load carriage. *Journal of neuroengineering and rehabilitation* **11**, 1–11 (2014).

[S2] I. Kang, H. Hsu, A. Young, The effect of hip assistance levels on human energetic cost using robotic hip exoskeletons. *IEEE Robotics and Automation Letters* **4**, 430–437 (2019).

[S3] J. Kim, G. Lee, R. Heimgartner, D. Arumukhom Revi, N. Karavas, D. Nathanson, I. Galiana, A. Eckert-Erdheim, P. Murphy, D. Perry, others, Reducing the metabolic rate of walking and running with a versatile, portable exosuit. *Science* **365**, 668–672 (2019).

[S4] W. Cao, C. Chen, H. Hu, K. Fang, X. Wu, Effect of hip assistance modes on metabolic cost of walking with a soft exoskeleton. *IEEE Transactions on Automation Science and Engineering* **18**, 426–436 (2020).

[S5] S. Galle, P. Malcolm, S. H. Collins, D. De Clercq, Reducing the metabolic cost of walking with an ankle exoskeleton: interaction between actuation timing and power. *Journal of neuroengineering and rehabilitation* **14**, 1–16 (2017).

[S6] L. Chen, C. Chen, Z. Wang, X. Ye, Y. Liu, X. Wu, A novel lightweight wearable soft exosuit for reducing the metabolic rate and muscle fatigue. *Biosensors* **11**, 215 (2021).

[S7] L. N. Awad, J. Bae, K. O'donnell, S. M. De Rossi, K. Hendron, L. H. Sloot, P. Kudzia, S. Allen, K. G. Holt, T. D. Ellis, others, A soft robotic exosuit improves walking in patients after stroke. *Science translational medicine* **9**, eaai9084 (2017).

Comfort evaluation questionnaire

Participants completed a post-trial comfort questionnaire following treadmill walking with and without the R-HEXsuit. The questionnaire consisted of ten items designed to assess perceived safety, resistance, comfort, fit, weight, and usability of the device. articipants were asked to rate their agreement with the following statements:

1. "I felt safe when the device was working as I walked"
2. "I felt the device offered me resistance when walking"
3. "I felt the device did not affect the movement of my legs when I walked"
4. "I felt unsafe when the device was worn and activated"
5. "I felt confident when using the device"
6. "The weight of the device was acceptable"
7. "I felt that the size and fit of the device was appropriate for me"
8. "The device was comfortable when worn and activated"
9. "I felt the device stayed in position once it had been fitted and during the investigation"
10. "I would be willing to wear this device for longer periods if needed"

Each response was given on a 5-point Likert scale ranging from Strongly disagree (1) to Strongly agree (5).
